# Inverse Problem in Nondestructive Testing Using Arrayed Eddy Current Sensors

**DOI:** 10.3390/s100908696

**Published:** 2010-09-20

**Authors:** Abdelhalim Zaoui, Hocine Menana, Mouloud Feliachi, Gérard Berthiau

**Affiliations:** 1 Electromagnetic Systems Laboratory, EMP, BP-17 Bordj El Bahri, 16111 Algiers, Algeria; 2 IREENA, 37, Boulevard de l’université, BP-406, 44602 Saint-Nazaire Cedex, France; E-Mails: hocine.menana@univ-nantes.fr (H.M.); mouloud.feliachi@univ-nantes.fr (M.F.); gerard.berthiau@univ-nantes.fr (G.B.)

**Keywords:** arrayed eddy current sensor, superposition principle, ideal crack model, reciprocity principle, inverse problem, genetic algorithms

## Abstract

A fast crack profile reconstitution model in nondestructive testing is developed using an arrayed eddy current sensor. The inverse problem is based on an iterative solving of the direct problem using genetic algorithms. In the direct problem, assuming a current excitation, the incident field produced by all the coils of the arrayed sensor is obtained by the translation and superposition of the 2D axisymmetric finite element results obtained for one coil; the impedance variation of each coil, due to the crack, is obtained by the reciprocity principle involving the dyadic Green’s function. For the inverse problem, the surface of the crack is subdivided into rectangular cells, and the objective function is expressed only in terms of the depth of each cell. The evaluation of the dyadic Green’s function matrix is made independently of the iterative procedure, making the inversion very fast.

## 1. Introduction

**Figure 1.**
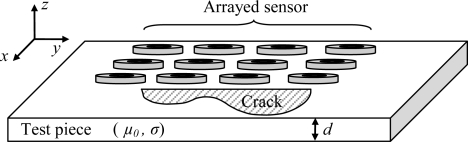
An arrayed eddy current sensor above a piece with a crack.

The use of arrayed eddy current (EC) sensors in Non Destructive Testing (NDT) provides high speed inspection and better space resolution by miniaturization of their coils. The arrayed sensors can make a measurement of large surfaces without a scan, as illustrated in the Figure 1, which results in a gain in time and measurement noise reduction; on the other hand, compared to conventional EC sensors, arrayed EC sensors provide more information about the defect characteristics.

**Figure 2.**
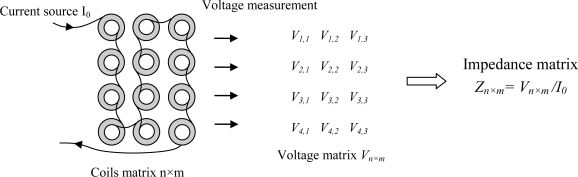
Impedance matrix measurement.

There are several configurations of arrayed eddy current sensors [1–3]; when their coils are fed separately, the effect of the adjacent coils is negligible; the modeling approach is then the same as for a single coil sensors. In this work, we consider an arrayed sensor in which the coils are connected in series and fed simultaneously by a current source as shown in Figure 2. The advantages of such configuration are:

- The synchronization of the supply and the measurement is not required for the electronic component.
- The measurement of the coils impedance is carried throw the voltage measurement.
- The incident electric field on the scan surface is uniform because the coils are connected in series, and this is independent of the work piece surface state.

The investigation is done by the measurement of the impedance variation of each coil. The purpose is to determine a crack shape and size using the measurements provided by such a sensor in a real time investigation.

The inversion method proposed is based on the iterative solving of the direct problem; it is thus important to have a fast tool to solve the latter. The use of the 3D finite element method would be very expensive in terms of memory space and CPU time. On the other hand, the analytic models lack the flexibility to handle complex geometries. In this work, we use the ideal crack model [4–6], generalized to arrayed eddy current sensors [7]. In the ideal crack model, the effect of the crack is represented by a current dipole layer on its surface, evaluated by an integral equation involving the electric dyadic Green’s functions and the normal incident electric field on the crack surface. The impedance variation of each coil is evaluated using the reciprocity principle. The evaluation of the dyadic Green’s function matrix is made independently of the iterative procedure of inversion; this makes the inversion to be very fast. On the other hand, a fast calculation of the incident field, produced by all the coils of the arrayed sensor, on the crack surface is achieved by making a translation and a superposition of the 2D axisymmetric finite element results obtained for one coil [7].

## 2. The Modeled System

**Figure 3.**
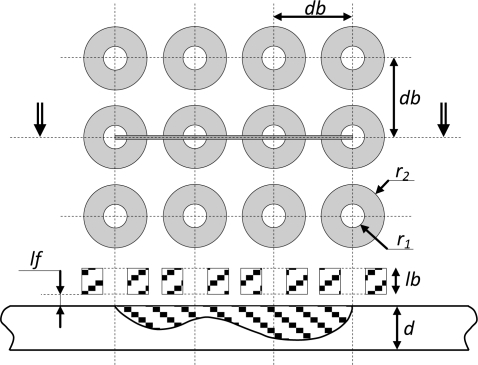
The modeled system.

Figure 3 describes the modeled system. It is constituted of a (3 × 4) matrix of identical coils situated above a conductive plate characterized by a conductivity σ and the free space permeability *μ_0_*. The plate contains an ideal crack of a surface *S* with an arbitrary shape described in Figure 3. The arrayed sensor coils are fed in series by a current source with a time harmonic variation $i_{s}(t)=\sqrt{2}I_{s}e^{j\omega t}$. Table 1 gives the numerical values of the fixed parameters of the system [9].

## 3. Direct Problem Formulation

The direct problem is based on the generalization of the ideal crack model to an arrayed eddy current sensor [7], which we recall briefly in this section. Firstly, the electric field induced by a single coil in the unflawed piece is calculated using the 2D axisymmetric finite element method. The electromagnetic problem formulation is given by (1), involving the magnetic vector potential *A* and the current source density *Js*. The total electric field *E^T^* induced by all the coils constituting the arrayed sensor is then obtained by (2), making a spatial translation and a superposition of the results obtained for the single coil [8]. In (2), *Aφ* is the magnetic vector potential solution of (1) for one coil, *nc* is the number of coils constituting the arrayed sensor, “*ox_k_*, *oy_k_*” are the center coordinates of the coil *k*, “*x,y,z*” are the Cartesian coordinates of the computing point, *r_k_* is the distance between the computing point and the axis of the coil *k*, and *sign(I_sk_)* indicates the direction of the current in the coil *k*.

(1) $$\left(\frac{\partial^{2}}{\partial r^{2}}+\frac{\partial^{2}}{\partial z^{2}}+\frac{\partial }{r\partial r}-\frac{1}{r^{2}}-j\omega \mu \sigma \right)A_{\phi }=\mu J_{s}\;,$$

(2) $$\{\begin{array}{l} E_{x}^{T}(x,y,z)=-j\omega \sum_{k=1}^{nc}\mathrm{sign}(I_{\mathrm{sk}})\frac{oy_{k}-y}{r_{k}}A_{\phi }(r_{k},z),\\ E_{y}^{T}(x,y,z)=-j\omega \sum_{k=1}^{\mathrm{nc}}\mathrm{sign}(I_{\mathrm{sk}})\frac{x-ox_{k}}{r_{k}}A_{\phi }(r_{k},z),\\ E_{z}^{T}(x,y,z)=0. \end{array}$$

Once the total normal incident field $E_{N}^{T}$ on the surface *S* of the ideal crack is determined, we calculate an equivalent current dipole *p* normal to this surface *S* by using the following integral equation [9]:

(3) $$E_{n}^{T}(r_{0})-\underset{r\to r_{0}}{\text{lim}}\left\{j\omega \mu_{0}\int_{S}G^{\mathrm{nn}}(r,r^{\prime })p(r^{\prime })ds\right\}=0;r_{0}\in S\;,$$

where:

(4) $$G^{\mathrm{nn}}(r,r^{\prime })=\hat{n}.\bar{G}(r,r^{\prime }).\hat{n}.$$

In (4), *n̑* is the vector normal to the surface *S*, and *Ḡ* (*r*, *r*′) is the electric dyadic Green function satisfying Equation (5) and subjected to the same continuity conditions as the electric field. In (5), *Ī* = *x̂x̂* + *ŷŷ* + *ẑẑ* is the unit tensor and *k*^2^ = *jωμ*_0_*σ*:

(5) $$\nabla \times \nabla \times \bar{G}(r,r^{\prime })-k^{2}\bar{G}(r,r^{\prime })=\bar{I}\delta (r-r^{\prime })$$

The integral equation (3) is solved using the moment method. The crack surface is subdivided into (*N* *=* *n_L_* × *n_d_*) rectangular elements of equal surfaces *S_e_*; the dipole density is considered constant in each element. We obtain the following matrix equation:

(6) {E}=[G]{P}.

The vectors *E* and *P* are of dimension (*N*); containing respectively the values of $E_{n}^{\left(k\right)}$ and *p* for the *N* elements of the crack grid. The matrix *G* is of dimension (*N* × *N*); its elements are calculated as follows:

(7) $$g(i,j)=\int_{s_{j}}G^{\mathrm{nn}}(r_{i},r_{j})dS,\;\;\;(i=1,..N,j=1,..N).$$

Using the reciprocity principle, the impedance variation of a coil k of the arrayed sensor is given by the following equation:

(8) $$\Delta Z^{\left(k\right)}=-\frac{1}{I_{s}^{2}}\int_{s}E_{n}^{\left(k\right)}p\;ds$$

In (6) $E_{n}^{\left(k\right)}$ and *p* are scalars representing, respectively, the part of the normal electric field induced by the coil *k* on the surface *S*, and the normal current dipole solution of (3). The discrete form of (8) is given by:

(9) $$\Delta Z^{\left(k\right)}=-\frac{S_{e}}{I_{s}^{2}}\sum_{i=1}^{N}E(i)P(i).$$

## 4. Inverse Problem

### 4.1. Reference data

The reference data for the inversion are obtained by a 3D finite element computation code developed in our laboratory. The computation code is based on the AV-A formulation [10] associated to the Gmsh meshing software [11]. We obtained the following impedance variation matrix, representing the impedances variations of the (3 × 4) matrix of coils constituting the arrayed EC sensor:

$$\left|\Delta Z*|\;=\left[\begin{array}{cccc} -0.0008-0.0025i & 0.0008+0.0008i & 0.0009+0.0040i & -0.0020-0.0005i\\ -0.0464-0.0125i & -0.1204+0.0755i & -0.1783+0.1374i & -0.0835-0.0002i\\ -0.0008-0.0024i & 0.0007+0.0008i & 0.0008+0.0041i & -0.0019-0.0005i \end{array}\right](\Omega \right)$$

**Figure 5.**
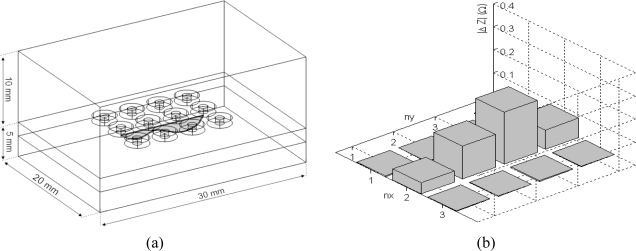
**(a)** The 3D finite element modeled geometry. **(b)** The obtained impedances variations.

Figures 5a and 5b represent the 3D finite element modeled geometry and the 3d plot of |ΔZ*| respectively; the latter gives an overview of the crack profile.

### 4.2. Inversion procedure

**Figure 6.**
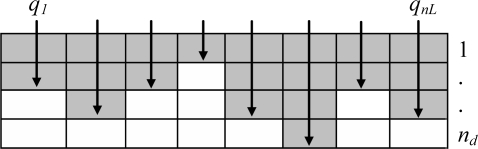
Example of a crack shape defined by the discrete values *q_i_*.

The detection of the crack is observed through the variation of the impedance matrix. In the initial step, we don’t know the exact position and orientation of the crack under the arrayed sensors. The adjustment of the position of the latter by looking for the maximum variation of the matrix impedance is necessary with the aim of getting the crack in the middle and on the main axis of the arrayed sensor. This manual operation makes the inverse problem easier and reduces it to the determination of the crack profile. It is assumed that the crack is embedded in a known rectangular area of dimensions *L×d*. This rectangle is subdivided into *N=n_L_×n_d_* rectangular cells. The crack profile is described by a vector *q* containing *n_L_* integer numbers varying between *0* and *n_d_*. An example of an arbitrary crack shape representation using these discrete values is given in Figure 6. The objective function is expressed as follows:

(10) $$ɛ(q_{i})=\sum_{k=1,nc}||\Delta Z_{k}(q_{i})-\Delta Z_{k}^{*}|{|}_{*},q_{i}\in N,0\leq q_{i}\leq n_{d},i=1...n_{L}$$

The norm used here is the absolute value which takes less computation time than the square root norm. For a better consideration of the real part in the minimization of the objective function, we separate it from the imaginary part in the impedance variation as follows:

(11) $${\left\|\Delta Z_{k}(q_{i})-\Delta Z_{k}^{*}\right\|}_{*}=\left|\frac{\text{Re}\left(\Delta Z_{k}(q_{i})-\Delta Z_{k}^{*}\right)}{\text{Re}\left(\Delta Z_{k}^{*}\right)}\right|+\left|\frac{\text{Im}\left(\Delta Z_{k}(q_{i})-\Delta Z_{k}^{*}\right)}{\text{Im}\left(\Delta Z_{k}^{*}\right)}\right|$$

We use the genetic algorithm for the minimization of the objective function (11). Genetic algorithms have been widely used, associated to the finite element method for the optimization of electromagnetic devices [12,13]. It is based on the principle of natural selection. A set of potential solutions (a population) is obtained against fitness criteria, and, through iterations, is refined with mutation and recombination [14].

**Figure 7.**
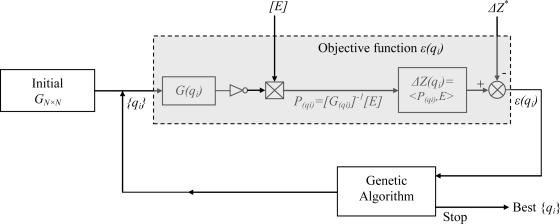
The inversion flow chart.

In the first step, we suppose that the crack occupies all the rectangle surface (*L* × *d*); the matrix *G*, as well as the normal incident field $E_{n}^{\left(k\right)}$, are then calculated once for all the cells of the grid (*n_L_* × *n_d_*) independently of the inversion procedure which is explained in Figure 7. It is based on an iterative solution of only (6) and (9). In each iteration, the elements *q_i_* of the vector *q* are obtained by the genetic algorithm; the matrix *G*, as well as the vector $E_{n}^{\left(k\right)}$, are then actualized by eliminating the rows and the columns corresponding to the cells which do not belong to the new crack surface; once the actualized matrix *G(q_i_)* is inverted, the vector *P* is then recalculated by (7) and *ΔZ_k_* is reevaluated by (9). Finally, an optimal vector *q* is obtained; the values of its elements determine the discrete shape of the crack. This procedure is very fast since the *q_i_* are integer values belonging to a short interval [*0 n_d_*].

**Figure 8.**
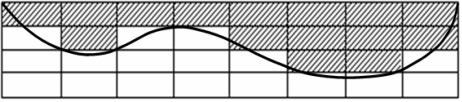
Inversion results *q = [1 2 1 1 2 3 3 2]*.

For the considered example we have chosen the discretization (*n_L_* × *n_d_* *= 8* × *4*); the variable *q_i_* can take four values (1, 2, 3 and 4) corresponding respectively to 25, 50, 75 and 100% of the plate thickness. For a fast computation, the variable *q_i_* is coded on two Boolean variables (00, 01, 10 and 11). The objective function in finality depends on *2* × *n_L_* *= 16* Boolean variables which correspond in the software implementation to the dimension of one short integer variable. We used the genetic algorithm toolbox of Matlab (*gatools* function) with the following parameters:

In Figure 8 we present the inversion results *q* (hatched cells) obtained for the considered example. Since the inversion method is heuristic, the execution time varies from 10 to 40 seconds on a 3.4 GHz Pentium 4 PC. The number of generations varies between 50 and 100 depending on the initial solution.

The part of computation done by the genetic algorithms is not time consuming according to their simple operation. The most consuming time in this inversion is the evaluation of the objective function and the inversion of the reduced matrix *G(q_i_)*.

## 5. Conclusions

We have presented a fast crack profile reconstitution procedure using arrayed eddy current sensor data. The use of the reciprocity and superposition principles allows a fast resolution of the direct problem. In the inverse problem, which is based on an iterative solving of the direct one, we adopted a coarse approximation of the crack profile which is represented by only a few discrete values; this makes the inversion procedure very fast when using genetic algorithms. This method can give a real time inspection when implemented in an embedded NDT hardware.

## Figures and Tables

**Figure 4.**
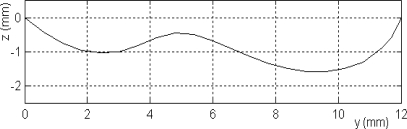
Crack shape.

**Table 1.** The Fixed parameter of the modeled system.

**Parameters**		**Values**
Frequency:		300 kHz
Coils:	Inner radius, *r_1_*	0.6 mm
	Outer radius, *r_2_*	1.6 mm
	Height, *lb*	0.8 mm
	Lift-off, *lf*	0.5 mm
	Number of turns, *N*	140
	Distance between the coils, *db*	4 mm
Plate:	Thickness, *d*	2 mm
	Conductivity, *σ*	1 MS/m
Crack:	Length, *L*	12 mm
	Thickness	0.2 mm
	Depth	Arbitrary shape (Figure 3)

**Table 2.** The Fixed Parameters for the Genetic Algorithm.

**Parameters**	**Values**
Population :	64
Crossover rates (Uniform) :	0.8
Mutation rates (Heuristic) :	0.02
